# Effects of Oxidative Stress on the Solubility of HRD1, a Ubiquitin Ligase Implicated in Alzheimer’s Disease

**DOI:** 10.1371/journal.pone.0094576

**Published:** 2014-05-01

**Authors:** Ryo Saito, Masayuki Kaneko, Yoshihisa Kitamura, Kazuyuki Takata, Koichi Kawada, Yasunobu Okuma, Yasuyuki Nomura

**Affiliations:** 1 Department of Pharmacology, Faculty of Pharmaceutical Sciences, Chiba Institute of Science, Choshi, Japan; 2 Research Fellow of Japan Society for the Promotion of Science, Tokyo, Japan; 3 Laboratory of Medical Therapeutics and Molecular Therapeutics, Gifu Pharmaceutical University, Gifu, Japan; 4 Department of Clinical and Translational Physiology, Kyoto Pharmaceutical University, Kyoto, Japan; 5 Department of Pharmacology, Kurume University School of Medicine, Kurume, Japan; Hokkaido University, Japan

## Abstract

The E3 ubiquitin ligase HRD1 is found in the endoplasmic reticulum membrane of brain neurons and is involved in endoplasmic reticulum-associated degradation. We previously demonstrated that suppression of HRD1 expression in neurons causes accumulation of amyloid precursor protein, resulting in amyloid β production associated with endoplasmic reticulum stress and apoptosis. Furthermore, HRD1 levels are significantly decreased in the cerebral cortex of Alzheimer’s disease patients because of its insolubility. The mechanisms that affect HRD1 solubility are not well understood. We here show that HRD1 protein was insolubilized by oxidative stress but not by other Alzheimer’s disease-related molecules and stressors, such as amyloid β, tau, and endoplasmic reticulum stress. Furthermore, we raise the possibility that modifications of HRD1 by 4-hydroxy-2-nonenal, an oxidative stress marker, decrease HRD1 protein solubility and the oxidative stress led to the accumulation of HRD1 into the aggresome. Thus, oxidative stress-induced HRD1 insolubilization might be involved in a vicious cycle of increased amyloid β production and amyloid β-induced oxidative stress in Alzheimer’s disease pathogenesis.

## Introduction

Alzheimer’s disease (AD), a progressive neurodegenerative disorder, is the most common cause of dementia in the elderly. The well-known neuropathological hallmarks of AD are extracellular senile plaques, which are aggregates of toxic amyloid β (Aβ) peptides, such as Aβ40 and Aβ42. Aβs, Aβ40 and Aβ42, are peptides generated from the amyloid precursor protein (APP) through sequential proteolytic cleavages by β-secretase (BACE1) and γ-secretase complexes [1], [2]. APP, a type I transmembrane glycoprotein with a short intracellular carboxyl terminus, is folded and *N*-glycosylated in the endoplasmic reticulum (ER). The “*Swedish*” *APP* double mutation results in Aβ overproduction, causing early onset familial AD [3]. Aggregated Aβ peptides form characteristic senile plaques in the brain tissues of AD patients, whereas intracellular neurofibrillary tangles (NFTs) are composed of paired helical filaments of hyperphosphorylated microtubule-associated protein tau [4] in affected cortical and subcortical neurons. A number of *tau* mutants, such as P301L, V337M, and R406W, accelerate the aggregation of tau into filaments [5]. Tau pathology is a later event in AD progression, probably triggered by Aβ-dependent hyperphosphorylation of tau [6]. Toxic Aβ peptides and hyperphosphorylated tau both interfere with numerous neuronal functions, such as ER function and intracellular trafficking of proteins [7].

ER plays a key role in protein synthesis; newly synthesized membrane and secretory proteins mature in ER, through protein processing, glycosylation, and disulfide bond formation. Various stresses, including hypoxia, glucose starvation, and viral infection, affect ER function and lead to ER stress, which is characterized by the accumulation of unfolded proteins in the ER. Under such conditions termed ER stress, a series of signaling pathways, the unfolded protein response (UPR), including ER chaperone induction and ER-associated degradation (ERAD) are activated in response to unfolded proteins accumulated in the ER [8], [9]. In the ERAD pathway, which is an ER protein quality control system and a defense mechanism against ER stress, ERAD target proteins are removed from the ER by retrograde transport to the cytosol, where they are degraded by the ubiquitin–proteasome system [10], [11].

Our recent studies have implicated ER stress and ERAD dysfunction in AD pathogenesis [2]. The E3 ubiquitin ligase HRD1, which is a human homolog of yeast Hrd1p/Der3p, forms a complex with its stabilizing factor SEL1L, a human homolog of yeast Hrd3p, in the ER membrane [12]. Furthermore, in the brain, *HRD1* is only expressed in neurons, and not glia [13]. In addition, HRD1 is also localized to neural stem/progenitor cells in the subventricular zone of the adult mouse [14]. *HRD1* and *SEL1L* expression induced by ER stress play major roles in ERAD and protect against ER stress-induced apoptosis [15], [16]. HRD1 is involved in the degradation of 3-hydroxy-3-methylglutaryl coenzyme A reductase, CD-3δ, TCR-α, p53, and several neurodegenerative disease-related proteins, such as Parkin-associated endothelin receptor-like receptor, prion protein, and huntingtin protein [17]–[21]. Misfolded MHC class I heavy chain, Nrf1, and Z variant α1-antitrypsin are also identified as a substrate for HRD1 [22], [23].

We recently demonstrated that *HRD1* expression promotes the ubiquitination and degradation of unfolded APP in the ERAD pathway, resulting in decreased Aβ production. Conversely, suppression of *HRD1* expression leads to APP accumulation and Aβ production in neurons. In addition, we found that AD-affected neurons are under ER stress and, a significant decrease in HRD1 levels in the NP-40-soluble fraction was observed in the cerebral cortex of AD patients [2], which negatively correlated with Aβ accumulation levels in the human cerebral cortex [24]. Furthermore, we found an increase in the HRD1 levels in the NP-40-insoluble fraction, suggesting protein insolubilization [25]. Because the mechanism(s) underlying the change in HRD1 solubility are unclear, we have investigated the possible roles of AD-related molecules and stresses, such as Aβ, tau, ER stress, and oxidative stress.

## Materials and Methods

### Antibodies and Chemicals

Primary antibodies with the following specificities were used: HRD1/SYVN1 (C-term; Sigma-Aldrich, St. Louis, MO, USA), Sel1L (T-17; Santa Cruz Biotechnology), protein disulfide isomerase (PDI; RL90; Thermo Scientific Pierce Products), tau (Tau-5; Millipore, Bedford, MA, USA), γ-tubulin (GTU-88; Sigma-Aldrich), β-actin (C4; Santa Cruz Biotechnology), 4-hydroxy-2-nonenal (4-HNE; HNEJ-2; JaICA, Shizuoka, Japan). Conjugated secondary antibodies were anti-rabbit and anti-mouse immunoglobulin G (IgG)–horseradish peroxidase (HRP) (GE Healthcare, Buckinghamshire, UK), anti-goat IgG–HRP (Promega, Madison, WI, USA). Alexa Fluor 488- and Alexa Fluor 546-conjugated secondary antibodies were purchased from Molecular Probes (Eugene, Oregon, USA). Thapsigargin, tunicamycin, and hydrogen peroxide (H_2_O_2_) were purchased from Wako Pure Chemical Industries (Osaka, Japan). Rotenone, HNE-DMA, and G418 were purchased from Sigma-Aldrich.

### Transgenic Mouse Brain Samples

Transgenic mice expressing hemizygous human Swedish double mutated *APP* (*APP_SWE_: Tg2576*) or the four-repeat isoform of human *tau* (*Tau_P301L_: JNPL3*) were purchased from Taconic (Germantown, NY, USA) and maintained in a room at 22–24°C under a constant 12 h light/12 h dark cycle. All animal experiments were performed in accordance with NIH Guidelines for Care and Use of Laboratory Animals and approved by the Committee for Animal Research at Kyoto Pharmaceutical University. Each mouse was euthanized by cervical spine dislocation without anesthesia, and subsequently the brain tissue was carefully isolated.

### Cell Culture

Mouse neuroblastoma Neuro2a (N2a) and human neuroblastoma SH-SY5Y cells were maintained in DMEM (Sigma-Aldrich) supplemented with 10% (v/v) heat-inactivated FBS (BioWest, Nuaillé, France) at 37°C in 5% CO_2_, 95% humidified air. N2a cells stably expressing *presenilin 2* (*PS2*) were maintained in DMEM under constant G418 (Geneticin) selection.

### Plasmids

Expression vectors for wild-type (wt) human *APP_695_* (tagged with FLAG and 6×His epitopes at the C terminus), human *PS2*, and the human *tau* isoforms *four-repeat tau* (*0N4R tau*) and *P301L* were gifts of Dr. Toshiharu Suzuki (Hokkaido University, Japan), Dr. Takeshi Iwatsubo (University of Tokyo, Japan), and Dr. Tomohiro Miyasaka (Doshishya University, Japan), respectively.

### Cycloheximide Assay

Normal SH-SY5Y cells were pretreated with 25 µg/ml cycloheximide (CHX) for 5 min before treated with or without 100 µM H_2_O_2_. Cells were harvested at 6 hours after a treatment of H_2_O_2_.

### Protein and mRNA Analysis

For protein analysis, cells were solubilized in Tris buffer containing protease inhibitors [10 mM Tris-HCl (pH 7.6), 420 mM NaCl, 1 mM EDTA, 1% NP-40, 10 µg/ml aprotinin, 10 µg/ml leupeptin, 1 mM phenylmethylsulfonyl fluoride (PMSF), 100 mM sodium orthovanadate, 10 mM sodium fluoride (NaF), and 100 nM okadaic acid]. Pellets of NP-40-insoluble proteins were collected by centrifugation for 20 min at 20,400 ×*g* and re-extracted in 10 mM Tris-HCl (pH 8.0), 420 mM NaCl, 1 mM EDTA, 1% Triton X-100, 0.5% sodium deoxycholate, 0.1% sodium dodecyl sulfate, 10 µg/ml aprotinin, 10 µg/ml leupeptin, 1 mM PMSF, 100 mM sodium orthovanadate, 10 mM NaF, and 100 nM okadaic acid. Unbroken fractions were removed by centrifugation for 20 min at 20,400 ×*g*. Protein samples were analyzed by western blotting using a LAS-3000 luminescent image analyzer (Fujifilm, Tokyo, Japan). Quantitative analysis was performed using Multi Gauge software (Fujifilm).

For RNA analysis, cells were homogenized in TRI reagent (Sigma-Aldrich) and reverse transcribed into cDNA using SuperScript VILO cDNA synthesis kit (Invitrogen). Gene expression was analyzed in duplicate with the TaqMan-based real-time PCR assay using a 7500 Real-Time PCR System (Applied Biosystems, Foster City, CA, USA), as described previously [12].

### Immunoprecipitation

Endogenous HRD1 was immunoprecipitated from SH-SY5Y cells after lysis in buffer [10 mM Tris-HCl (pH 7.6), 420 mM NaCl, 1 mM EDTA, 1% NP-40, 10 µg/ml aprotinin, 10 µg/ml leupeptin, and 1 mM PMSF]. Supernatant containing 3 mg total protein was incubated overnight at 4°C with 3 µg/ml anti-HRD1/SYVN1 antibody (C-term; Sigma-Aldrich) or 3 µg/ml normal rabbit IgG (Millipore). Supernatants were incubated with Protein G Magnetic Beads (Millipore) for 2 hours, and the beads were rinsed three times with wash buffer [10 mM Tris-HCl (pH 7.6), 150 mM NaCl, and 0.1% Triton X-100]. Immune complexes were eluted with 0.1 M glycine-HCl (pH 3.0)] and then incubated for 5 min on ice. Extracts were neutralized with 1 M Tris-HCl (pH 8.0) and incubated with or without 100 µM 4-HNE at 37°C for 3 h. Total cell extracts and immunoprecipitates were treated with β-mercaptoethanol-free Laemmli SDS-PAGE sample buffer and analyzed by western blotting using ECL Prime Western Blotting Detection Reagent (GE Healthcare).

### Immunocytochemistry

SH-SY5Y cells were incubated overnight at 4°C with anti-HRD1/SYVN1 (diluted 1∶100 in PBS) and anti-γ-tubulin (diluted 1∶100 in PBS) antibodies, followed by Alexa Fluor 546-conjugated anti-rabbit and Alexa Fluor 488-conjugated anti-mouse antibodies (diluted 1∶200 in PBS) for 30 min at room temperature. After washing with PBS, these cells were mounted on to glass slides using a SlowFade Gold antifade reagent (Invitrogen). Immunostained cells were visualized using an LSM 510 META confocal microscope (Carl Zeiss AG, Oberkochen, Germany).

### ELISA for Aβ Peptides

Secreted Aβ peptides were measured by a standard sandwich ELISA using a human Aβ (1–40) and (1–42) assay kit from IBL (Takasaki, Japan). For APP overexpression, N2a cells stably expressing *PS2* were transfected with *APP-FLAG* using Lipofectamine LTX reagent (Invitrogen).

### Statistical Methods

Results were compared using two-tailed Student’s *t*-test or two-tailed multiple *t*-test with Bonferroni correction following analysis of variance. All data are expressed as mean ± standard error of mean. A *p* value less than 0.05 was considered statistically significant.

## Results

### HRD1 Solubility in Neurons was Independent of Aβ Peptide or Tau Levels

**Figure 1:**
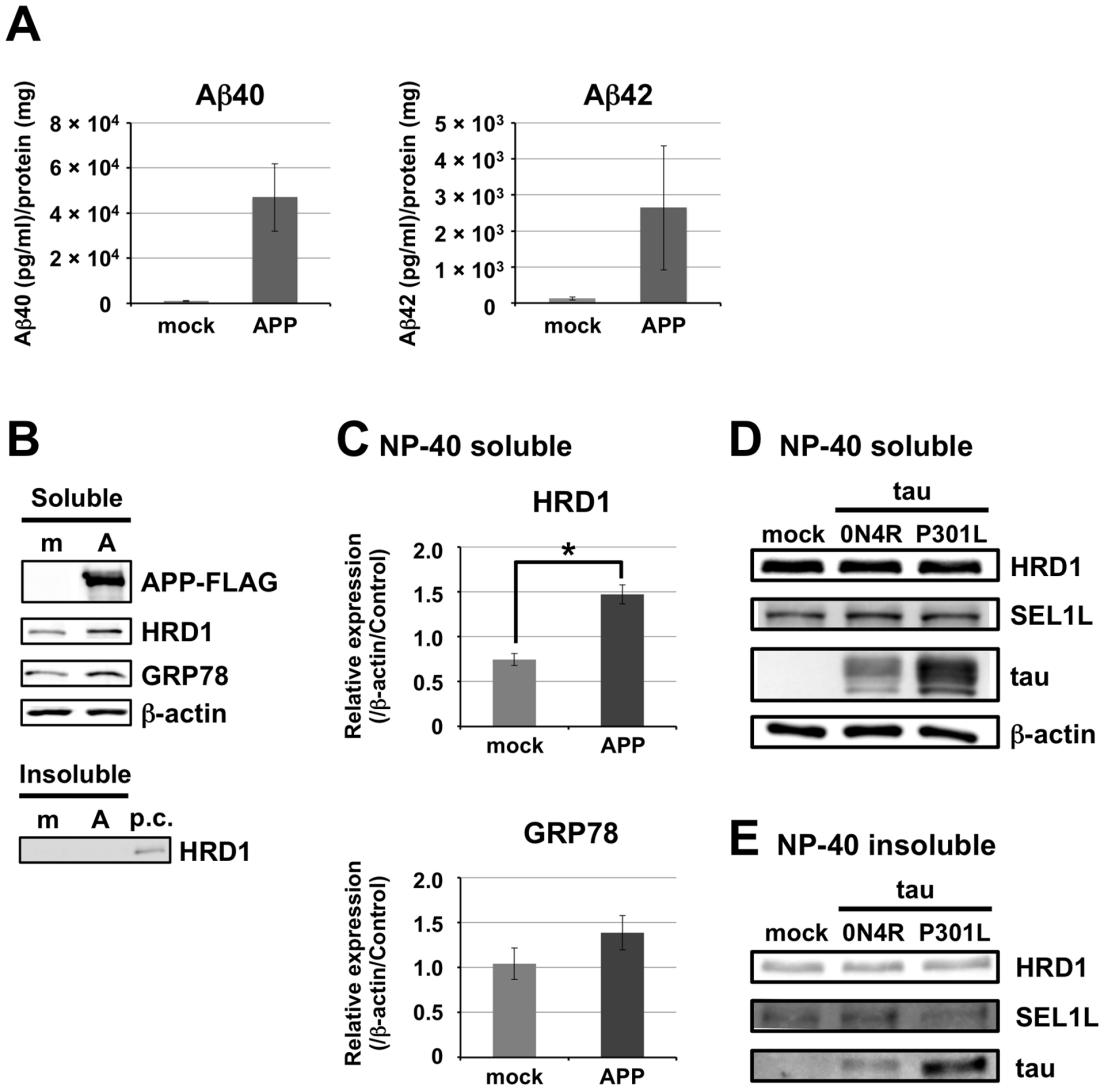
Effect of Aβ and tau on HRD1 solubility in N2a cells. *A*. ELISA of Aβ peptides, Aβ40 and Aβ42, in N2a cells stably expressing PS2 24 h after transfection with *APP-FLAG* or an empty vector (mock). Results are expressed as a ratio of Aβ peptide (pg/ml) to whole cell protein extract (mg). *B*. Total cell lysates of NP-40-soluble and -insoluble fractions analyzed by western blotting with the indicated antibodies. *C*. Statistical analysis of (*B*) NP-40 soluble HRD1. Data are normalized to β-actin levels, and results are expressed as the fold increase compared with protein expression levels in untransfected control (mean ± SEM; *n* = 3). Statistical analysis was performed with ANOVA, followed by Student’s *t*-test (mock vs. APP; *p<0.05). D-E. Western blotting of (*D*) NP-40-soluble and (*E*) NP-40-insoluble proteins in the total cell lysates of N2a cells 24 h after transfection with the four-repeat isoform of human tau (tau_0N4R_; 0N4R), mutant tau (tau_P301L_; P301L), or the empty vector (mock). Abbreviation: m, mock. A, APP. p.c., positive control, which was extracted by NP-40 from wild-type HRD1-expressing Flp-In-293 cell.

We previously showed that HRD1 levels in the NP-40-soluble fraction and Aβ peptide accumulation are correlated in the human cerebral cortex. However, it is unclear whether Aβ generation is dependent on HRD1, or whether HRD1 is decreased by Aβ-induced neurotoxicity. In addition, although Aβ peptide accumulation in the cerebral cortex is a pathological hallmark of AD, the mechanism of Aβ toxicity remains unclear. To determine whether HRD1 solubility was affected by Aβ, we transiently overexpressed APP in N2a cells stably expressing PS2 to promote Aβ production and found increased Aβ peptide secretion; approximately 50-fold for Aβ40 24 h after transfection, and approximately 20-fold for Aβ42 (Fig. 1A). However, no change in the HRD1 levels was observed in the NP-40-insoluble fraction (Fig. 1B). These results indicate that HRD1 solubility was unrelated to the Aβ level. On the other hand, the HRD1 levels in the NP-40-soluble fraction were significantly increased in APP-overexpressing cells (Fig. 1B, C). Additionally, because the GRP78 levels also tend to increase in the NP-40-soluble fraction, it seems that the APP-overexpressing cells were under mild ER-stress conditions (Fig. 1B, C).

The accumulation of hyperphosphorylated tau is believed to play a crucial but undefined role in AD pathogenesis. We assessed the possible effects of tau on HRD1 solubility in N2a cells using transient overexpression of tau 0N4R (wt tau containing 4 *C*-terminal repeat tau but with no *N*-terminal insert) or the tau mutant P301L. The level of these tau proteins was increased in the NP-40-soluble and -insoluble fractions compared with that in N2a cells transfected with an empty vector (mock). However, HRD1 and SEL1L levels in the NP-40-soluble and -insoluble fractions were unchanged (Fig. 1D, E). These results show that the accumulation of soluble or insoluble tau had no effect on HRD1 and SEL1L solubility.

### HRD1 Solubility was Unaffected by Aβ and Tau Overexpression in Transgenic Mice

**Figure 2:**
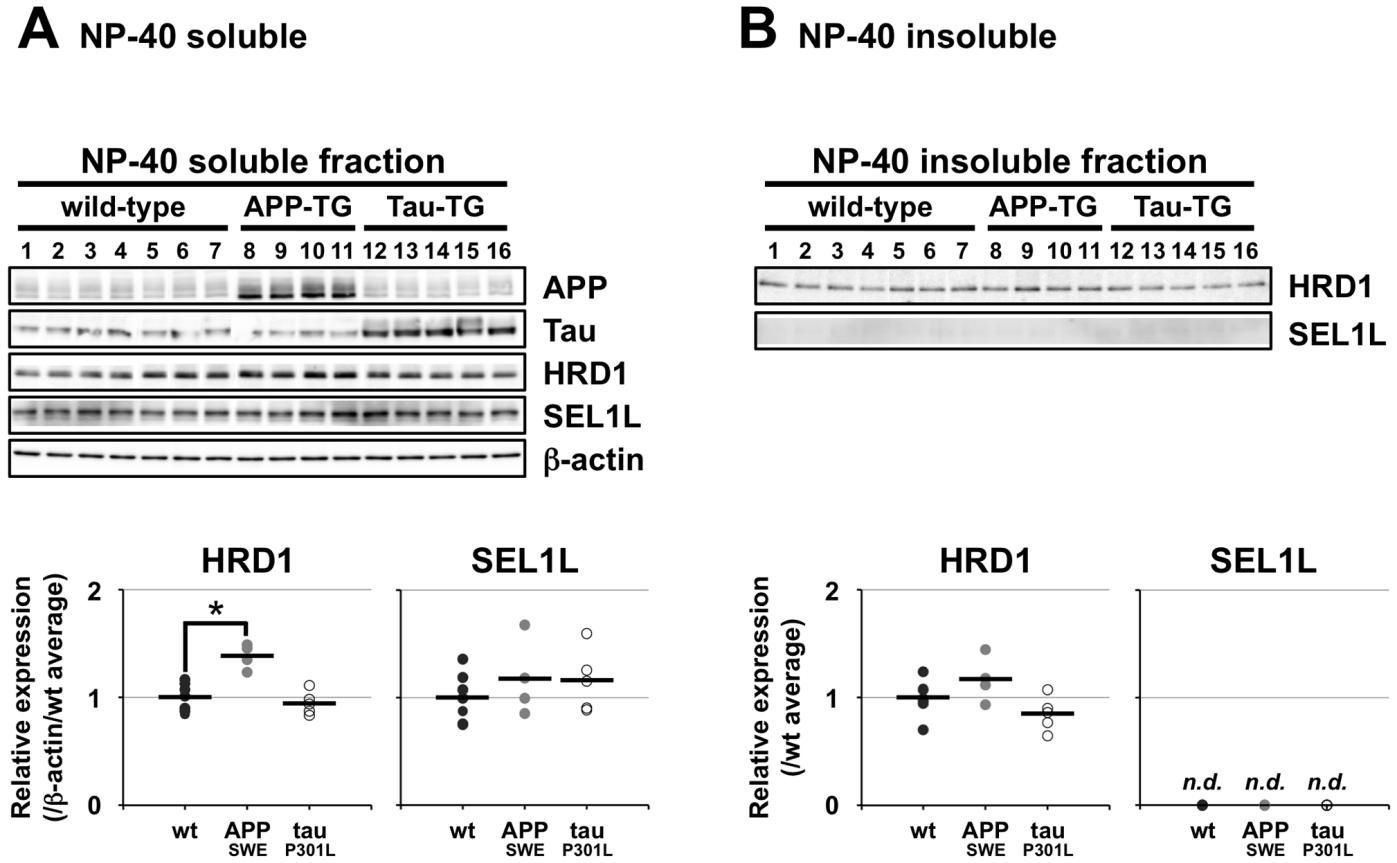
Influence of Aβ or tau on HRD1 and SEL1L solubility in the cerebral cortex of Tg2576 or JNPL3 mice. HRD1 and SEL1L levels in the cerebral cortex of Tg2576 (hemizygous human Swedish double-mutated APP transgenic; APP_SWE_) or JNPL3 (human mutated four-repeat tau_P301L_ transgenic; Tau_P301L_) mice. The cerebral cortex of each mouse at 16 to 20 months of age was examined. The total lysates of (*A*) NP-40-soluble and (*B*) -insoluble fractions were analyzed by western blotting with the indicated antibodies; statistical analysis of these results are expressed as a dot plot (• wt, *n* = 7; • APP_SWE_, *n* = 4; ○ Tau_P301L_, *n* = 5). Data are normalized to the wt averages for each fraction. Statistical analysis was performed with ANOVA, followed by Bonferroni correction (Control vs. APP_SWE_ and Tau_P301L_; *p<0.05).

Short-term overexpression of Aβ or tau had no effect on HRD1 or SEL1L solubility *in vitro*. However, HRD1 solubility might be affected over a longer period. We investigated this possibility by assaying HRD1 levels in the cerebral cortex of hemizygous *APP_SWE_* transgenic mice, which express the Swedish form of human *APP* (Tg2576), and in Tau_P301L_ transgenic mice, which express the four-repeat isoform of human tau (JNPL3). Tg2576 mice are an AD model that develops amyloid plaques but without neurodegenerative changes, whereas JNPL3 is a model of tauopathy that develops NFTs with neuronal loss [26]. Consistent with our *in vitro* data (Fig. 1), HRD1 and SEL1L levels in the NP-40-insoluble fraction were not decreased in these transgenic mice compared with the wt controls (Fig. 2A, B). However, the HRD1 levels in the NP-40-soluble fraction was significantly increased in Tg2576 mice (Fig. 2A), consistent with the experimental result from APP and PS2-overexpressing N2a cells (Fig. 1B, C). These results indicate that HRD1 might act as a defensive factor against APP- and/or Aβ-induced neurotoxicity.

### ER Stress did not Affect HRD1 Solubility in Neurons

**Figure 3:**
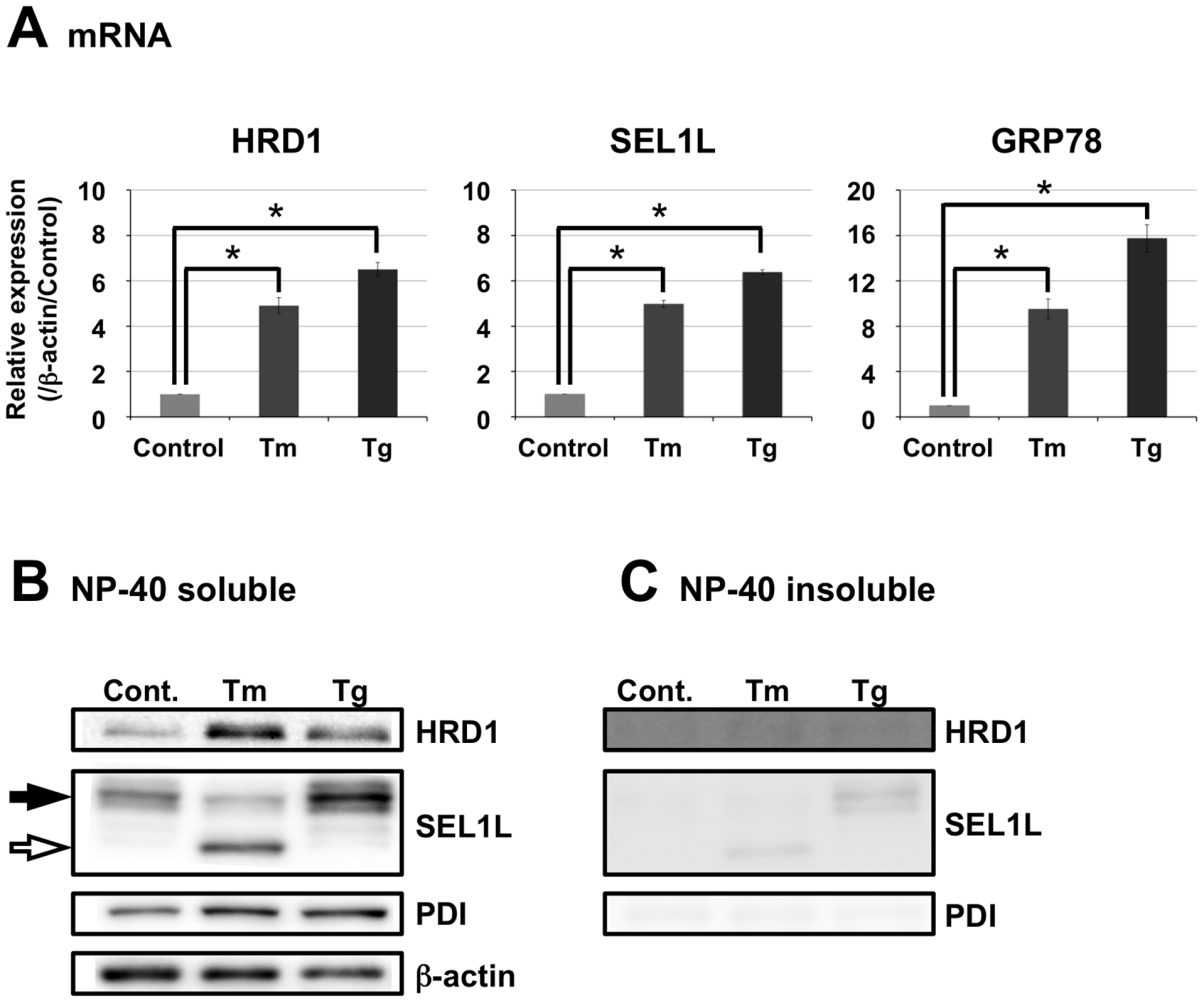
Induction of HRD1 and SEL1L expression by ER stress-inducing agents. *A*. Real-time PCR analysis of gene expression in N2a cells treated for 24 h with or without tunicamycin (Tm; an inhibitor of *N*-linked glycosylation) and thapsigargin (Tg; an inhibitor of Ca^2+^ ATPase). Data are normalized to the amount of β-actin; results are expressed as a fold increase compared with the non-treated control (mean ± SEM; *n* = 3). Statistical analysis was performed with ANOVA, followed by Bonferroni correction (Control vs. Tm and Tg; *p<0.05). *B-C.* Western blotting of (*B*) NP-40-soluble and (*C*) -insoluble fractions from N2a cells treated with or without Tm and Tg for 24 h using the indicated antibodies. The black arrow in soluble fraction indicates mature SEL1L, and the white arrow indicates immature SEL1L without glycosylation.

We previously demonstrated that HRD1 levels in the cerebral cortex of AD patients was significantly decreased by insolubility and that AD brains were under ER stress [2], [25]. Therefore, we next investigated the effect of ER stress on HRD1 solubility in N2a cells using the ER stress inducers tunicamycin (an inhibitor of protein *N*-linked glycosylation) and thapsigargin (an inhibitor of the ER Ca^2+^-ATPase). Expression of *HRD1*, *SEL1L*, and *GRP78* mRNAs, as well as HRD1, SEL1L, and PDI levels in the NP-40-soluble fraction, were all significantly increased by tunicamycin or thapsigargin (Fig. 3A, B). However, no significant changes in the HRD1, SEL1L, and PDI levels were observed in the NP-40-insoluble fraction from tunicamycin- or thapsigargin-treated cells (Fig. 3C). These results suggest that although HRD1 protein was induced by ER stress, this did not affect HRD1 solubility.

### Oxidative Stress Induced HRD1 Insolubility

**Figure 4:**
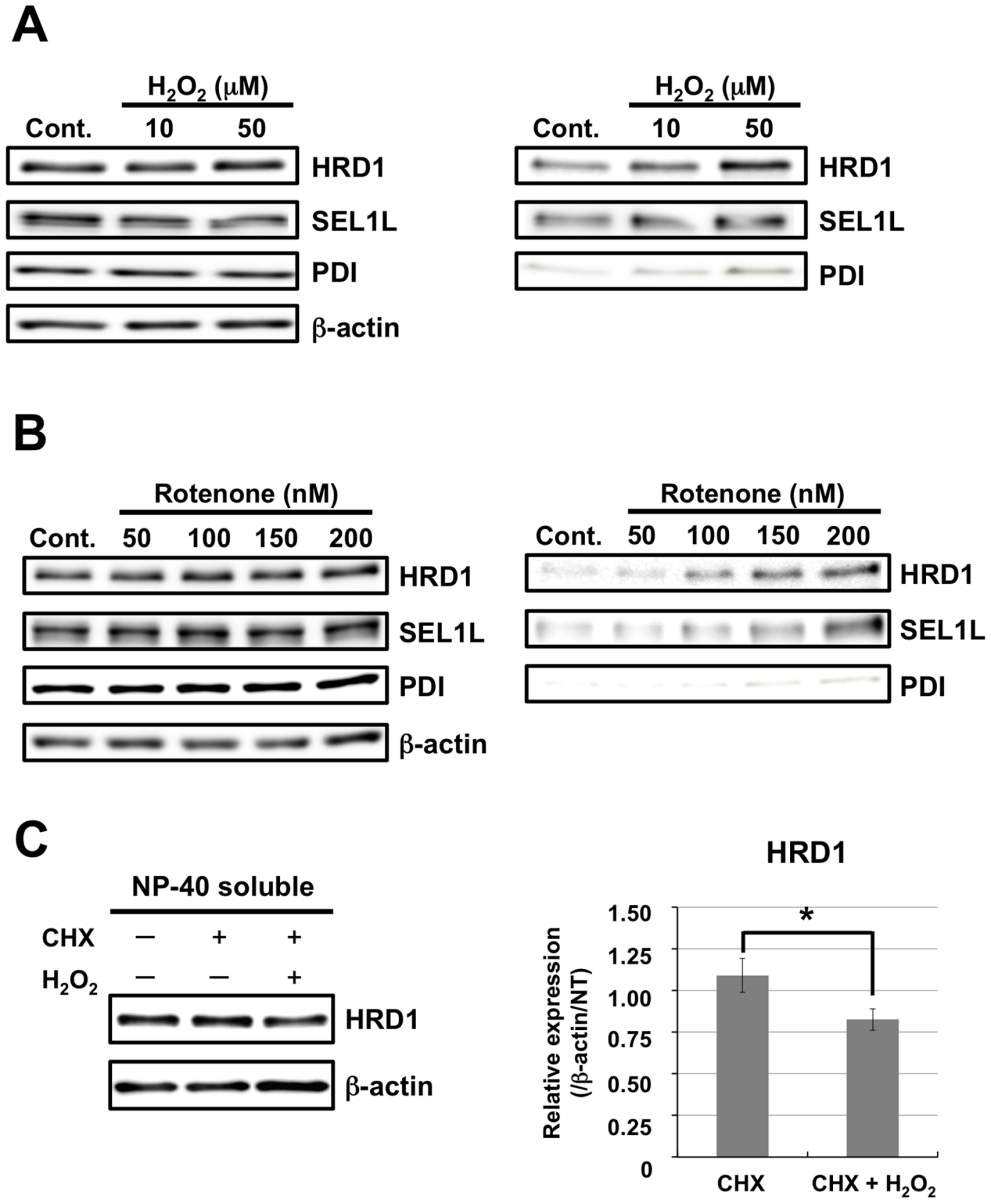
Insolubilization of HRD1 and SEL1L proteins by oxidative stress-inducing agents. *A–C.* SH-SY5Y cells were treated with or without H_2_O_2_ (*A*) and rotenone (*B*) for 24 h. The total cell lysates of NP-40-soluble (left panel) and -insoluble (right panel) fractions were analyzed by western blotting with the indicated antibodies. *C*. Decrease in soluble HRD1 due to oxidative stress under inhibition of *de novo* protein synthesis. SH-SY5Y normal cells were treated with 25 µg/ml CHX. At 5 min after treatment, the cells were additionally treated with or without 100 µM hydrogen peroxide (H_2_O_2_) for 6 h. The total cell lysates of NP-40 soluble fractions were analyzed by western blotting using the indicated antibodies. Data are normalized to the amount of β-actin; results are expressed as a fold increase compared with the non-treated control (mean ± SEM; *n* = 6). Statistical analysis was performed with ANOVA, followed by Student’s *t*-test (CHX vs. CHX+H_2_O_2_; *p<0.05). Abbreviation: NT, non-treated control.

Aβ deposition occurs in blood vessels in the vicinity of cortical micro-infarcts. However, it remains unclear whether and how oxidative stress from chronic cerebral ischemia or cerebrovascular accident is involved in AD pathogenesis [27]. To investigate the influence of oxidative stress on HRD1 and SEL1L solubility, we exposed human neuroblastoma SH-SY5Y cells to exogenous oxidative stress using H_2_O_2_. We found that this stress increased HRD1 and SEL1L levels in the NP-40-insoluble fraction in an H_2_O_2_ concentration-dependent manner, whereas their levels in the NP-40-soluble fraction were not significantly affected (Fig. 4A). Interestingly, there was no effect of H_2_O_2_ on PDI. We next exposed SH-SY5Y cells to endogenous oxidative stress using rotenone, an inhibitor of the mitochondrial electron transport chain and observed similar effects. The HRD1 and SEL1L levels in the NP-40-insoluble fraction were increased in a concentration-dependent manner, but those in the NP-40-soluble fraction remained unchanged (Fig. 4B). These results indicate that oxidative stress increases the HRD1 and SEL1L levels in the insoluble fraction.

### Soluble HRD1 Protein Levels were Maintained by *de novo* Protein Synthesis

In this study, we did not detect any decrease in HRD1 levels in the NP-40-soluble fraction due to any of the various stressors examined. Turnover of intracellular protein in most neuroblastoma cell lines is faster than in differentiated neurons; therefore, *de novo* protein synthesis in neuroblastoma cells might mask any decrease in soluble HRD1 protein due to insolubility. To test this hypothesis, we performed a cycloheximide chase assay. In the presence of cycloheximide, the soluble HRD1 protein levels were significantly decreased by H_2_O_2_ treatment (Fig. 4C). This result indicates that although oxidative stress actually reduced soluble protein levels, soluble HRD1 protein level was maintained by *de novo* protein synthesis in neuroblastoma cells.

### 4-HNE Modification of HRD1

**Figure 5:**
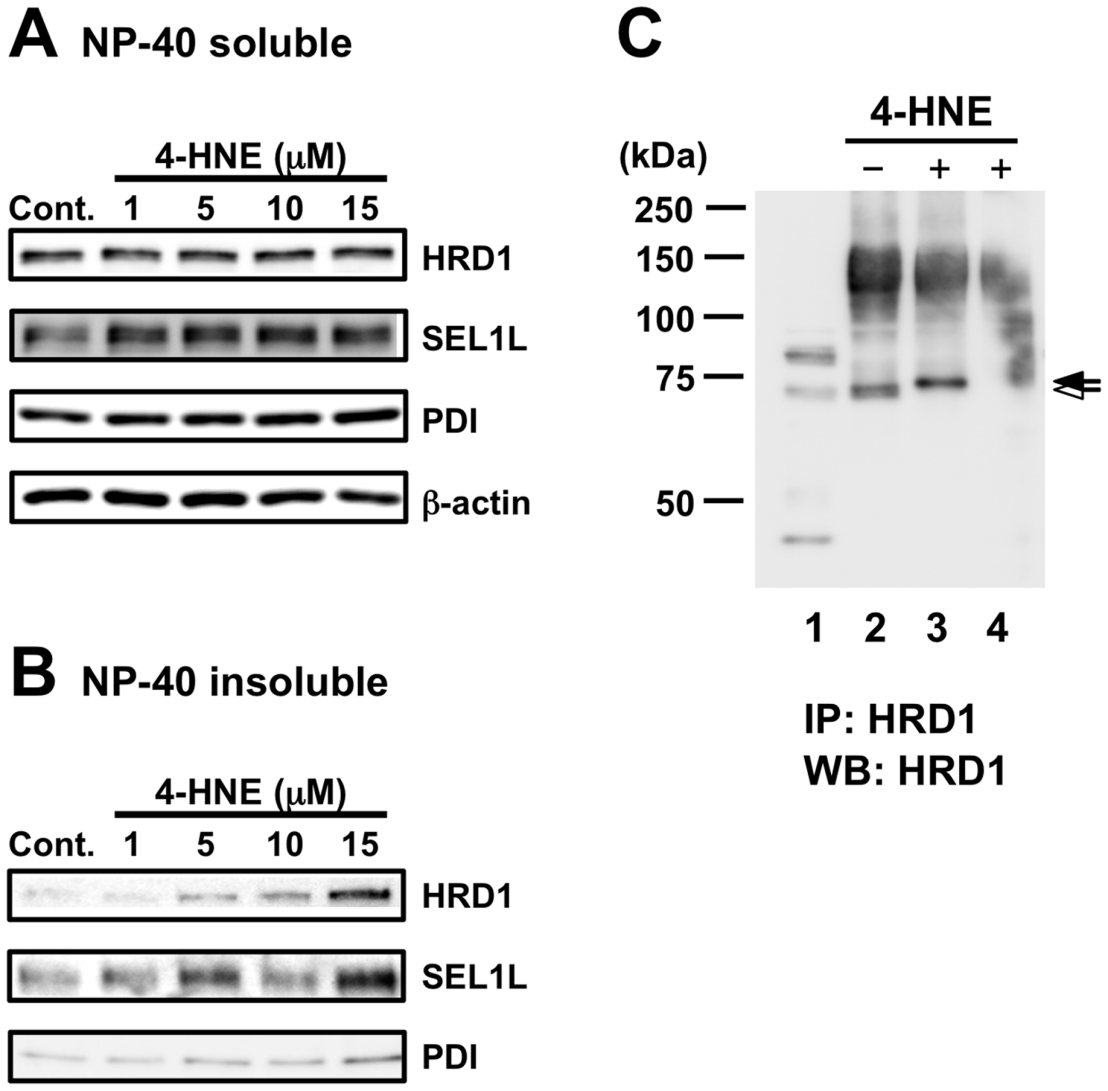
4-HNE modifications of HRD1 protein. *A–B*. SH-SY5Y cells were treated with or without 4-HNE for 24 h. The total cell lysates of NP-40-soluble (A) and -insoluble (B) fractions were analyzed by western blotting with the indicated antibodies. *C*. Total cell lysates were immunoprecipitated (IP) with anti-HRD1/SYVN1 antibody. These IP samples were treated with or without 100 µM of 4-HNE at 37°C for 3 h and then analyzed by western blotting with the indicated antibodies. Black arrow, 4-HNE-modified HRD1; white arrow, unmodified HRD1 protein. Lane 1, total cell lysate; Lane 2, IP with anti-HRD1/SYVN1 antibody; Lane 3, IP with anti-HRD1/SYVN1 antibody treated with 4-HNE; Lane 4, IP with anti-mouse IgG.

To further elucidate the influences of oxidative stress on HRD1 and SEL1L solubilization, SH-SY5Y cells were exposed to 4-HNE, an unsaturated aldehydic product of membrane lipid peroxidation that modifies cellular DNA and proteins and induces apoptosis [28]–[30]. Interestingly, the HRD1 and SEL1L levels in the NP-40-insoluble fraction were increased by exposure to 4-HNE (Fig. 5A), whereas those in the NP-40-soluble fraction remained unchanged (Fig. 5B). To investigate whether HRD1 protein were modified by 4-HNE, we exposed purified HRD1 to 4-HNE *in vitro* under non-reducing conditions and observed an increase in the molecular weight of HRD1 (Fig. 5C). This raises the possibility that 4-HNE modifications are involved in HRD1 solubility.

### Oxidative Stress Induced HRD1 Aggresome

**Figure 6:**
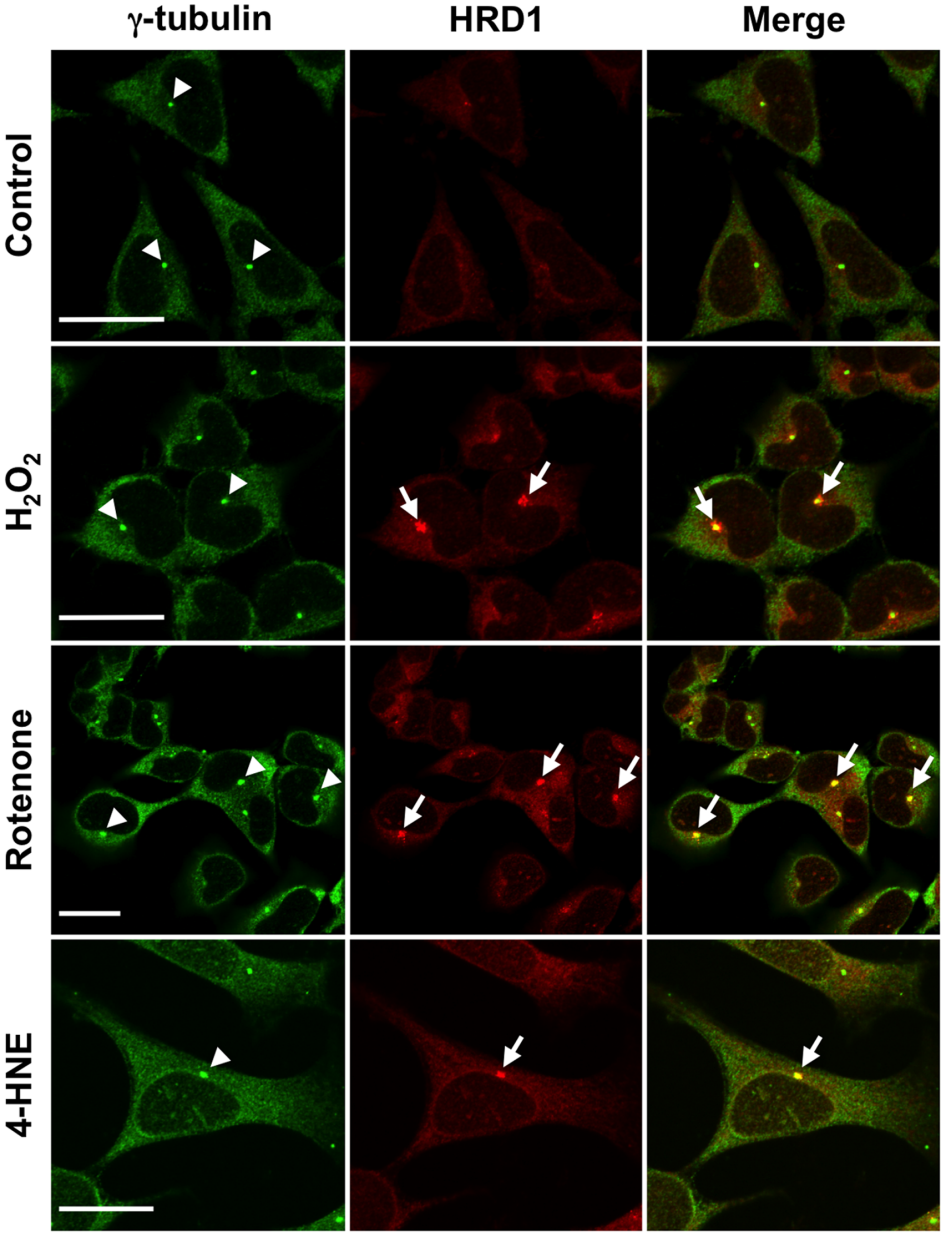
Induction of HRD1 aggresome formation by oxidative stress. SH-SH5H cells treated with H_2_O_2_ (10 µM), rotenone (10 nM), and 4-HNE (15 µM) for 48 h and then subjected to immunofluorescence staining using HRD1 (red) and γ-tubulin (green) antibodies. Arrowheads, MTOC; arrows, HRD1 in aggresomes. Scale bars, 20 µm.

It is generally accepted that protein aggregates, such as amyloid senile plaques, NFTs, and Lewy bodies, are insoluble. The aggresome is an aggregate of several denatured ER proteins exported to the microtubule-organizing center (MTOC), which is localized around the nucleus and mainly composed of γ-tubulin. To determine if insoluble HRD1 was a component of the aggresome, we examined HRD1 localization in cells under oxidative stress. Double immunofluorescence staining using anti-HRD1 and anti-γ-tubulin antibodies showed that HRD1 proteins were present in aggregates adjacent to ER following exposure to H_2_O_2_, rotenone, or 4-HNE (Fig. 6). In addition, the aggregated HRD1 protein was present near MTOC. Therefore, these results suggest that the insoluble HRD1 induced by the oxidative stressors was localized to the aggresome.

## Discussion

Our previous studies showed that HRD1 levels in the cerebral cortex inversely correlates with Aβ accumulation levels [24]. However, it is unclear whether the decrease in HRD1 protein causes Aβ generation or whether Aβ neurotoxicity decreases HRD1 levels. Recent reports showed that Aβ neurotoxicity-induced apoptosis is due to activation of ER stress-specific initiator caspases, including mouse caspase-12 and human caspase-4 [31], [32]. Furthermore, accumulation of Aβ in neurons could interfere with the ubiquitin–proteasome system by inhibiting the proteasome and deubiqitinases [33], [34]. Because HRD1 is a component of the ubiquitin–proteasome system, HRD1 may also be a target for interference by Aβ. In other words, HRD1 insolubility is likely to be caused by Aβ.

We here found that the Aβ level had no effect on HRD1 solubility in N2a cells stably expressing PS2. However, this experimental system is controversial, in part because the effects of Aβ on protein solubility may only become apparent over longer periods. Furthermore, we did not confirm whether the oligomeric and/or fibrillar Aβ were formed by endogenous Aβ. To solve these problems, we analyzed the cerebral cortex of the *APP_SWE_* transgenic mouse line Tg2576. Consistent with the *in vitro* results, insoluble HRD1 was not increased in the cerebral cortex of Tg2576 mice. In this study, we employed Tg2576 mice to evaluate the effect of accumulated Aβ without hyperphosphorylated tau and neurodegenerative change on HRD1 protein solubility. We found that other factors (besides accumulated Aβ) were necessary for the insolubilization of HRD1 protein in AD. These *in vivo* and *in vitro* results suggest the possibility that a decrease in HRD1 protein levels precedes Aβ accumulation in the cerebral cortex of AD patients.

Increased accumulation of hyperphosphorylated tau is also closely involved in AD pathogenesis. However, it is unclear whether phosphorylation and/or accumulation of tau affects ER protein stability. Our results here show that the ubiquitin ligase HRD1 and its stabilizing factor SEL1L, which are components of ERAD, were unaffected by the level of insoluble tau in neurons. Consistent with this, the HRD1 and SEL1 levels were normal in the cerebral cortex of Tau_P301L_ transgenic mice.

Ubiquitination of tau and phosphorylated tau (p-tau) by HRD1 targets these proteins for degradation by the proteasome [35]. Tau phosphorylation is mainly due to glycogen synthase kinase-3β (GSK-3β) [36], which is activated in relation to ER stress and the ER-associated chaperone Bip, also known as GRP78 [37]. Our results and these data suggest that dysfunction of the protein quality control in the ER leads to tau phosphorylation and accumulation. In addition, ER stress may occur in advance of tau hyperphosphorylation. Moreover, our analysis of the effects of Aβ and tau on HRD1 solubility supports the hypothesis that the HRD1 protein decreases before the increase in Aβ generation. However, it remains to be fully determined whether tau hyperphosphorylation leads to HRD1 insolubility because we did not assay hyperphosphorylated tau in our experiments. In addition, hyperphosphorylation of tau accumulated in the cerebral cortex of JNPL3 mice is at a low level. Regarding the interaction between HRD1 and p-tau [35], we believe that hyperphosphorylated tau is likely to lead to HRD1 accumulation and/or insolubility.

In a previous study, we found that the cerebral cortex of AD patients is under ER stress [2]. However, it remains unclear whether AD-related molecules, such as Aβ and hyperphosphorylated tau, cause ER stress, or whether they accumulate in response to ER stress. Our previous results showed that suppression of HRD1 expression causes APP accumulation and ER stress-induced apoptosis [2]. Based on this, we suggest that the decrease and insolubility of HRD1 precede Aβ accumulation and ER stress. In this study, we found that tunicamycin- and thapsigargin-induced ER stress had no effects on HRD1 solubility, although it did increase HRD1 protein expression, suggesting that HRD1 upregulated may protect against ER stress. Moreover, HRD1 upregulation did not affect its solubility, suggesting that increases in ER stress response-induced proteins do not provoke further ER stress but act to protect against it.

We found that insoluble HRD1 in neuronal cell lines was increased by oxidative stressors, such as H_2_O_2_, rotenone, and 4-HNE, resulting in the presence of HRD1 in aggresomes. In fact, increased oxidative stress is linked to AD progression. Furthermore, Aβ peptides are deposited in the vicinity of cortical micro-infarcts [27]. Although ischemia is known to cause oxidative stress, it is unclear how oxidative stress affects Aβ generation and thus AD onset. Our findings raise the possibility that the complex of the ubiquitin ligase HRD1 with SEL1L is a target of oxidative stress-inducing agents, such as reactive oxygen species and 4-HNE. Furthermore, these findings could provide important insights into the link between oxidative stress and Aβ generation. Thus, oxidative stress-induced inhibition of HRD1 may promote Aβ generation and accumulation.

Although we did not assess whether HRD1 enzyme activity was impaired by oxidative stress, we identified possible oxidative modification of the HRD1 protein. LC-MS/MS analysis revealed cysteine residues in the RING-finger domain of HRD1 as candidates for oxidative modification. In addition, 3D protein structure prediction of the RING-finger domain of HRD1 using the SWISS-MODEL (http://swissmodel.expasy.org) showed that the cysteine residues in RING are localized at the protein surface to coordinate zinc. Therefore, these cysteine residues in the RING of HRD1 may be subjected to oxidative modification. Furthermore, recent reports indicate that the cysteine residues in RING-finger domains of ubiquitin ligase are sensitive to nitrosative and oxidative modifications [38]. Furthermore, the enzyme activities of Parkin and XIAP (RING-type ubiquitin ligases, which are the same type of HRD1) are regulated by *S*-nitrosylation of the cysteine residues in the RING-finger domain [39]–[41]. Therefore, the enzyme activity of HRD1 may also be regulated by oxidative stressors though the oxidative modification of RING. In the present study, we found that the 4-HNE induced HRD1 insolubilization. It is well known that 4-HNE specifically modifies cysteine, histidine, and lysine residues [42]. Therefore, the 4-HNE-induced HRD1 insolubilization might be caused by 4-HNE modifications. In this study, we found the possibility that HRD1 protein was subject to 4-HNE modifications *in vitro*. Additionally, our recent study found that this 4-HNE-induced molecular shift in the HRD1 protein was almost completely prevented by *N*-ethylmaleimide, which causes irreversible alkylation of cysteine (data not shown). This raises the possibility that 4-HNE modifications are involved in HRD1 solubility. However, we have not identified 4-HNE modifications to the cysteine residues of HRD1 protein by LC-MS/MS analysis. This result of LC-MS/MS suggests the possibility that 4-HNE might be indirectly involved in oxidative modification of cysteine residues of HRD1 protein and affect HRD1 protein solubility. Because recent reports showed that 4-HNE induced oxidative stress though mitochondrial dysfunction [43], [44], 4-HNE-induced HRD1 insolubilization may be due to indirect oxidation as a result of mitochondrial dysfunction. Therefore, more detailed studies are needed to determine whether 4-HNE directly or indirectly modifies HRD1 and affects its solubility. On the other hand, the localization of HRD1 to the aggresome may indicate irreversible damage to HRD1 because the aggresome is mostly comprised of denatured proteins [45]. In general, it is believed that HRD1 localizes and functions in the ER, whereas denatured HRD1 is selectively removed from the ER via the ERAD pathway. Therefore, incorporation of HRD1 into aggresomes implies the loss of HRD1 function.

In this study, we demonstrated that *de novo* protein synthesis masks decrease in soluble HRD1 protein due to oxidative stress-induced insolubilization. Since AD patient’s brains appears to be exposed to various stress conditions, other stresses also implicated in soluble HRD1 depletion mechanism in addition to oxidative stress. For instance, ER stress response induces translational arrest. In fact, ER stress and protein synthesis inhibition were observed as AD pathological events [15], [40]. To elucidate more precise HRD1 insolubilization mechanism, it is necessary to investigate the effects of multiple stresses on HRD1 protein solubility.

We investigated age-related changes in soluble and insoluble HRD1 in the cerebral cortex of wt mice. We found that the level of soluble HRD1 protein remained constant with age, whereas insoluble HRD1 did not increase with age (Figure S1). These results suggest that HRD1 might be insolubilized by abrupt increases in oxidative stress, such as cerebral infarction, but not age-associated biological change.

Recent reports showed that Aβ enhances the ER stress response through mitochondrial dysfunction [41] and that ER–mitochondrial interactions are involved in Aβ-induced apoptosis [42]. Furthermore, oxidative stress triggered by mitochondrial dysfunction may be a key pathogenic trigger in AD pathogenesis [45]. Although these reports indicate a close relationship between Aβ and oxidative stress, HRD1 insolubility was only caused by oxidative stress. In this study, endogenous Aβ was insufficient to induce oxidative stress. Furthermore, because our experiments raised the possibility that HRD1 is a defensive factor against Aβ-induced neurotoxicity,oxidative stress-induced HRD1 insolubilization might interfere with ERAD systems, leading to a vicious cycle of increased Aβ production and Aβ-induced oxidative stress. Subsequently, accumulation of Aβ and ER stress promotes neurodegeneration and AD pathology. Our findings support a role for loss of HRD1-mediated ERAD in AD pathogenesis.

## Supporting Information

Figure S1 **Age-related changes in HRD1 protein solubility.** HRD1 in the cerebral cortex of 1.0, 1.5, and 2.0-year-old C57BL mice. The total lysates of NP-40-soluble (*A*) and -insoluble (*B*) fractions were analyzed by western blotting, quantified, and expressed as a dot plot.
(PDF)
