# Supplementary material for: Effects of Oxidative Stress on the Solubility of HRD1, a Ubiquitin Ligase Implicated in Alzheimer’s Disease
Source: PLoS One. 2014 May 1;9(5):e94576. doi: 10.1371/journal.pone.0094576 (PMC4006799; doi:10.1371/journal.pone.0094576)
Supplement: Figure S1 — Age-related changes in HRD1 protein solubility. HRD1 in the cerebral cortex of 1.0, 1.5, and 2.0-year-old C57BL mice. The total lysates of NP-40-soluble (A) and -insoluble (B) fractions were analyzed by western blotting, quantified, and expressed as a dot plot. (PDF) [file pone.0094576.s001.pdf]

Supporting Information

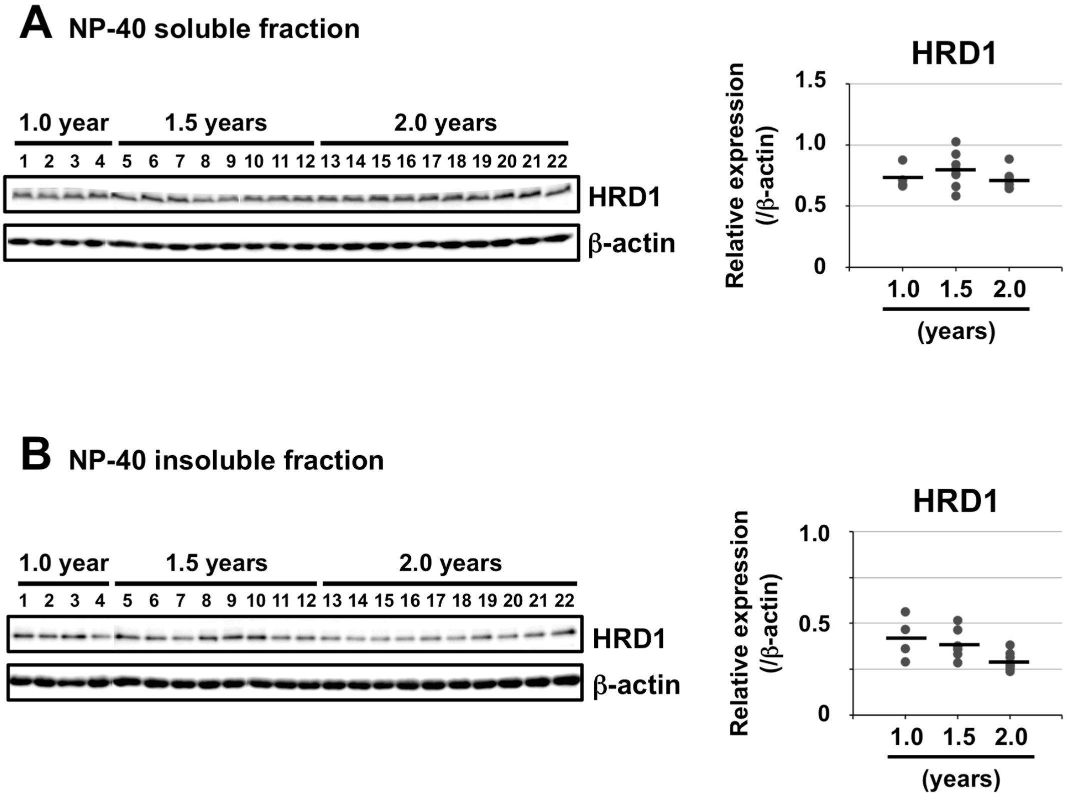

FIGURE S1. Age-related changes in HRD1 protein solubility. HRD1 in the cerebral cortex of 1.0, 1.5, and 2.0-year-old C57BL mice. The total lysates of NP-40-soluble (*A*) and -insoluble (*B*) fractions were analyzed by western blotting, quantified, and expressed as a dot plot.
